# Design of Reduction Process of SnO_2_ by CH_4_ for Efficient Sn Recovery

**DOI:** 10.1038/s41598-017-14826-7

**Published:** 2017-10-31

**Authors:** Hyunwoo Ha, Mi Yoo, Hyesung An, Kihyun Shin, Taeyang Han, Youhan Sohn, Sangyeol Kim, Sang-Ro Lee, Jun Hyun Han, Hyun You Kim

**Affiliations:** 1 Department of Materials Science and Engineering, Chungnam National University 99 Daehak-ro, Yuseong-gu, Daejeon, 34134 Republic of Korea; 2 Department of Materials Science and Engineering, KAIST, 291 Daehak-ro, Yuseong-gu, Daejeon, 34141 Korea; 3 A1 Engineering Co.,Ltd., 80-19 Yulchonsandan 1-ro, Haeryong-myeon, Suncheon-si, Jeollanam-do 58034 Republic of Korea; 4 Present Address: Department of Chemistry and the Institute for Computational Engineering and Sciences, University of Texas at Austin, Austin, TX USA

## Abstract

We design a novel method for the CH_4_ reduction of SnO_2_ for the efficient recovery of Sn from SnO_2_ through a study combining theory and experiment. The atomic-level process of CH_4_-SnO_2_ interaction and temperature-dependent reduction behavior of SnO_2_ were studied with a combination of a multi-scale computational method of thermodynamic simulations and density functional theory (DFT) calculations. We found that CH_4_ was a highly efficient and a versatile reducing agent, as the total reducing power of CH_4_ originates from the carbon and hydrogen of CH_4_, which sequentially reduce SnO_2_. Moreover, as a result of the CH_4_ reduction of SnO_2_, a mixture of CO and H_2_ was produced as a gas-phase product (syngas). The relative molar ratio of the produced gas-phase product was controllable by the reduction temperature and the amount of supplied CH_4_. The laboratory-scale experimental study confirmed that CH_4_ actively reduces SnO_2_, producing 99.34% high-purity Sn and H_2_ and CO. Our results present a novel method for an efficient, green, and economical recycling strategy for Sn with economic value added that is held by the co-produced clean energy source (syngas).

## Introduction

Recovering (extracting) metallic elements from ores has occurred throughout human history^1–5^. Advanced copper and iron smelting technology was required for the development of civilization in history^1–5^. However, although dry- or hydro-smelting technologies are currently used as a core technology in industry, many of these technologies are not green or environmentally friendly^6–13^. The most common dry-smelting or reduction of ores by carbon and flux typically byproduces CO_2_ and slag at high temperatures^13–15^. In addition, electrolytic smelting or refining of low-quality metal sources is not cost-effective and produces highly corrosive liquid wastes^6–10,16,17^. During the development of human civilization over the past thousands of years, the most easily mineable and accessible high purity ores have been used first due to economic efficiency. The relative depletion of most economically accessible and mineable ores naturally accompanies the accumulation of used metal wastes; many of these wastes are not appropriately recycled.

Sn (Tin) is a highly demanded industrial material^18–22^ that is important for the production of electronics^23–26^, sensors^27–30^, glasses^31–33^, and displays^23,34,35^. The industrial demand of Sn is expected to gradually increase in the near future^20–22^ as Sn plays as a central role in Pb-free solder^36,37^ and transparent electrode^23,34,35^. The current London metal exchange market price of Sn is approximately $19,900/metric ton as of July 2017^38^, which is more than 3 and 10 times more expensive than Cu and Al, respectively^38^. However, currently, only approximately 30% of the annual industrially consumed Sn is being recovered worldwide^39^, meaning that the remaining 70% of the Sn used is excluded from the recycling process and is eventually wasted. In principle, recovering a metallic element from used metals (wastes and scraps) requires a similar process to the initial ore smelting. To recover high-purity metal from used metal wastes, a combined smelting-electrolytic refining process is typically required, making the recovery process economically nonadvantageous^6,7,9,10,12,13,16^. Such a complicated recovery process weakens the economic driving force for the recovery of metals, such as Sn, which is consumed heavily worldwide.

Hydrogen or methane have been utilized as a reducing agent for metal oxides^40–47^. For example, methane reduction (MR)^41,46^ or hydrogen reduction of ZnO^44,48^ was proposed to overcome environmental or economical disadvantages of conventional dry-smelting or recovery techniques. To the best of our knowledge, there are few previous reports on the MR of SnO_2_. Eroglu and coworkers thermodynamically studied and experimentally demonstrated the feasibility of MR of SnO_2_ method^42^. They also utilized methane as a reducing agent of various metal oxides^43,45,49^ confirming the strong reducing power of methane. However, detailed atomic scale understanding of MR of SnO_2_, which is necessary for optimization of the MR reduction method, is scarce.

In this work, considering the findings of previous studies and combining the widely applied methane dry reforming (CH_4_ + CO_2_ → 2CO + 2H_2_)^50–53^ and the conventional reduction of Sn oxides by carbon (SnO_x_ + C → Sn + CO_x_), we study a novel, environmentally friendly MR method of SnO_2_. We hypothesized that carbon and hydrogen from methane independently and actively reduce SnO_2_, making the reduction process highly efficient. Moreover, because our MR method utilizes methane and SnO_2_ as a reducer and an oxidizer, respectively, the final gas-phase product naturally involves H_2_ and CO. The mixture of H_2_ and CO, syngas, can be utilized as a feedstock for further Fisher-Tropsch synthesis^50–53^ improving the economic accessibility of our method. Thermodynamic simulations confirmed the availability of the MR of SnO_2_ and deduced the optimal operation conditions for efficient Sn recovery and syngas (H_2_ + CO) production. Density functional theory (DFT) calculations revealed the atomic-level understanding of the process. Subsequent experiments demonstrated that the MR method is very promising for economic and environmentally friendly Sn recovery from SnO_x_-containing industrial wastes.

## Results

### Theoretical prediction of CH_4_ reduction of SnO_2_

**Figure 1:**
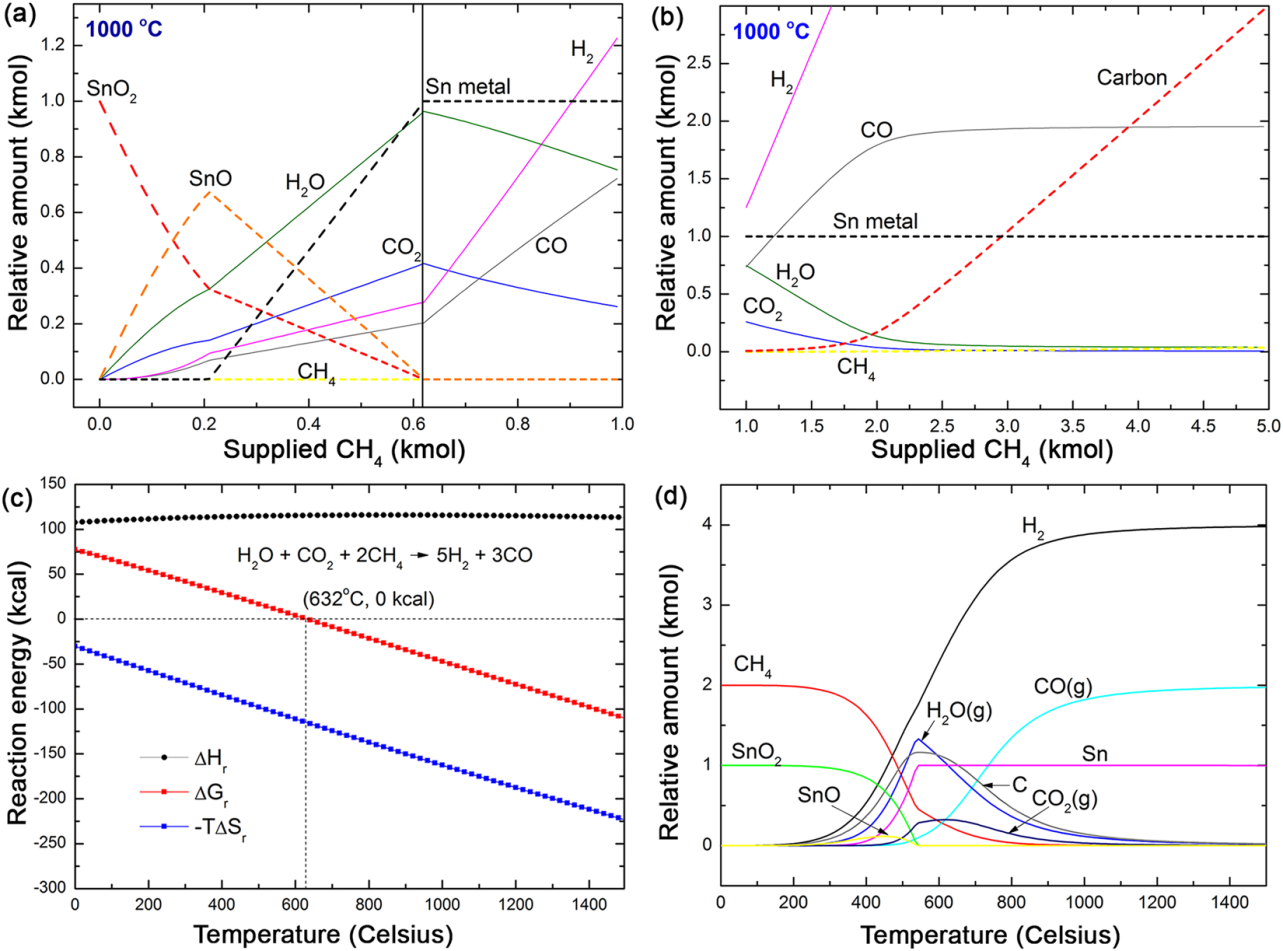
Theoretical prediction of the MR of SnO_2_. (**a**,**b**) Equilibrium concentration of the mixture of one kmole of SnO_2_ and *n*∙CH_4_ (*n* = 0~5, continuously increasing by a step of 0.01 kmole) at 1000 °C as a function of the amount of supplied CH_4_. (**a**) $0\le {\rm{n}}\le 1.0$, (**b**) between CH_4_ and the pre-produced gas-phase products occur as the R ratio exceeds 0.62. (**c**) Temperature dependent reaction energies of two sets of mixtures of gas-phase molecules. The red solid symbols represent the reaction Gibbs free energy, ΔG_r_, for H_2_O + CO_2_ + 2CH_4_ → 5H_2_ + 3CO. The gas phase reaction becomes thermodynamically driven at above 632 °C. (**d**) Temperature dependent equilibrium relative concentration of a SnO_2_-CH_4_ mixture. The initial R value was set to 2.0. Theoretical maximum recovery of Sn was achieved at approximately 550 °C. Although the solid-state reduction of SnO_2_ to Sn was completed at 550 °C, the relative concentration of the gas-phase products varies as a function of temperature and converges at approximately 1000 °C.

Figure 1a and b present the equilibrium concentrations of the mixture for a kmole of SnO_2_ and *n*∙CH_4_ (*n* = 0~5, continuously increasing by a step of 0.01 kmole) at 1000 °C as a function of the amount of supplied CH_4_. These diagrams were designed to phenomenologically describe the continuous reduction process that occurs inside the reduction furnace in which a certain amount of SnO_2_ is exposed to a stream of CH_4_. In the early phase of reduction, as the amount of supplied CH_4_ increases, SnO_2_ was gradually reduced to SnO rather than completely reduced to Sn. At less than R = 0.21 (${\rm{R}}={\rm{amount}}\,{\rm{of}}\,{\rm{supplied}}\,{{\rm{CH}}}_{4}/{\rm{amount}}\,{\rm{of}}\,{\rm{initial}}\,{{\rm{SnO}}}_{2}=0.21$), all the decreasing amount of SnO_2_ was reduced to SnO (Fig. 1a). In this early phase, the main gas-phase product was H_2_O.

As the amount of supplied CH_4_ exceeded R = 0.21, SnO_2_ and SnO both began to decreased producing the fully reduced metallic Sn (Fig. 1a). At an equilibrium condition, the supplied SnO_2_ can be completely reduced to metallic Sn at R = 0.62. Interestingly, even up to R = 0.62, H_2_O was the main gas-phase product. At R = 0.62, almost half the total supplied oxygen content from SnO_2_ was taken up by hydrogen (H_2_O), and the other half formed CO_2_ and CO. In the early phase of reduction at less than R = 0.62, the hydrogen and carbon from CH_4_ were independently acting as reducing agents. The solid-state reduction of SnO_2_ to SnO and Sn by CH_4_ was completed at R = 0.62. Up to this point, the amount of SnO_2_ and SnO was gradually decreasing and was finally reduced to metallic Sn.

As the R ratio exceeded 0.62, the gas-phase reactions occurred between the excess CH_4_ and the pre-existing H_2_, CO, O_2_, and H_2_O in an oxygen-depleted condition (Fig. 1b). All the oxygen from the SnO_2_ was already consumed by the hydrogen or carbon. The temperature dependent reaction Gibbs free energy, ΔG_r_, estimated for the reaction H_2_O + CO_2_ + 2CH_4_ → 5H_2_ + 3CO, show that ΔG_r_ becomes negative over at above 632 °C (Fig. 1c). A negative ΔG_r_ predicts that if CH_4_ is continuously supplied to the system over the equilibrium amount for complete solid-state SnO_2_ reduction to Sn (R = 0.62), the formation of H_2_ and CO becomes thermodynamically preferred at high temperature. As a result of this gas-phase reaction, a mixture of pre-existing CO_2_ and H_2_O and excessively supplied CH_4_ was converted to H_2_ and CO at a high temperature (1,000 °C) (Fig. 1b and c).

Figure 1d shows the temperature dependent equilibrium composition map of a mixture of SnO_2_ and CH_4_ with R = 2.0. This R value was the critical point where the gas-phase reaction described in Fig. 1c was almost completed. The MR of SnO_2_ begins at approximately 300 °C. In the temperature range between 300 °C and 550 °C, the sequential solid-state reduction of SnO_2_ to SnO and Sn was completed. As predicted in Fig. 1a and b, the amount of H_2_O, CO_2_, and solid-state carbon produced were rapidly increased in this temperature range. At greater than 550 °C, the gas-phase reaction described in Fig. 1c drives a redistribution of the gas-phase products. Because R = 2.0 is generally the condition with excess CH_4_, a mixture of CH_4_, H_2_O, and CO_2_ naturally transforms to H_2_ and CO. Moreover, solid-state carbon began to appear even in the initial phase of the solid-state reduction process due to the presence of excess CH_4_ in the reduction system. However, this carbon was also decreasing at temperatures greater than 550 °C at which the solid-state reduction is completed and the gas-phase reaction begins. At approximately 1000 °C, the entire gas-phase product was transformed to a mixture of H_2_ and CO, increasing the H_2_/CO ratio up to 2.15.

**Figure 2:**
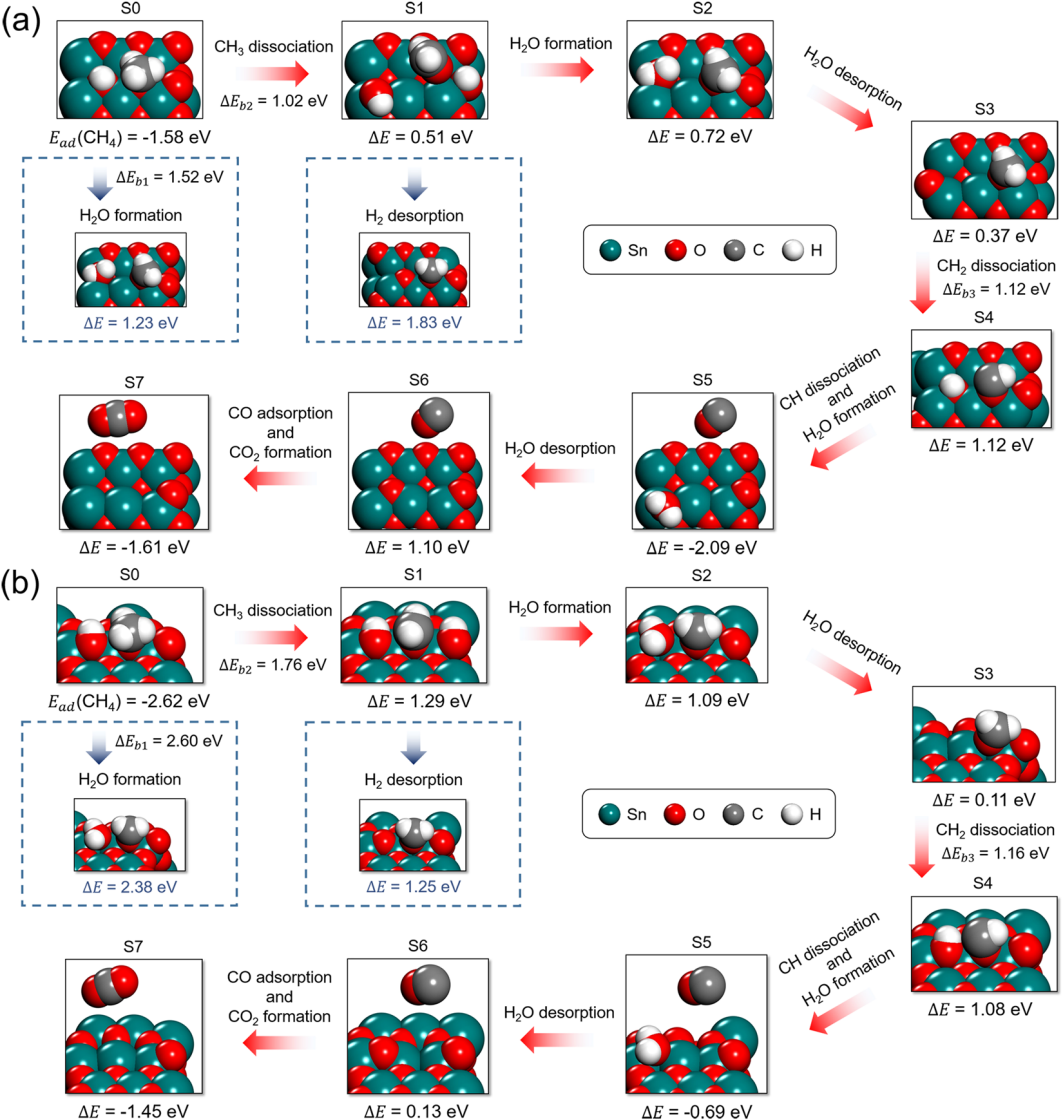
Energetics of SnO_2_ reduction by surface-bound CH_4_. (**a**) SnO_2_ (100) surface. (**b**) SnO_2_ (110) surface. On both SnO_2_ facets, formation of H_2_O was energetically preferred to H_2_. The red arrows present the preferred reaction pathway. *E*_ad_(CH_4_) represents the adsorption energy of CH_4_ on the SnO_2_ surfaces. Δ*E* of each step represents the energetic state of the current state relative to the previous state. For example, Δ*E* = 0.51 eV of S1 in (**a**) means that 0.51 eV of energy is required for CH_3_ dissociation from S0 to S1.

The molecular level process of SnO_2_-CH_4_ interaction was studied using DFT calculations. The DFT-calculated binding processes of CH_4_ on the (100) and (110) facets of the rutile-SnO_2_ show that SnO_2_ dissociatively binds CH_4_, producing a lattice oxygen-bound methyl group (O-CH_3_^*^) and a surface hydroxyl (-OH^*^) (S0 of Fig. 2a and b).

On SnO_2_(100) surface (Fig. 2a), the energetics shows that the direct production of H_2_O from O-CH_3_^*^ and –OH^*^ is highly endothermic (Δ*E* = 1.23 eV) and requires high energy barrier (Δ*E*_b1_ = 1.52 eV, a panel below the S0 of Fig. 2a). Oh the other hand, further dehydrogenation of O-CH_3_^*^ coupled with the formation of additional -OH^*^ is more energetically preferred (S1 of Fig. 2a, Δ*E* = 0.51 eV and Δ*E*_b2_ = 1.02 eV). We found that, from two separated –OH^*^ groups, the formation of H_2_O molecule is energetically preferred (S2 of Fig. 2) to the H_2_ formation (see a panel below S1 of Fig. 2a, Δ*E* = 1.83 eV) which is again highly endothermic. The desorption of water from the S2 requires only 0.37 eV (see S3 of Fig. 2a). However, further dehydrogenation of O-CH_2_^*^ group is endothermic and requires high barrier of 1.12 eV (S4 of Fig. 2a, Δ*E*_b3_ = 1.12 eV). Once a O-CH_2_^*^ acetylene group is dissociated, H_2_O and CO or CO_2_ production follows. Particularly, the preferred formation of H_2_O is thermodynamically and kinetically favored to the formation of H_2_, confirming the trend in Fig. 1a. Another interesting feature is that a CO molecule was spontaneously formed upon dehydrogenation of a O-CH^*^ group, as presented in S4 and S5 of Fig. 2a. We also found that this CO molecule directly attacks the surface and be transformed to CO_2_ with Δ*E* of −1.61 eV (S7, Fig. 2a). The processes presented in S5 to S7 suggest that solid carbon could directly reduce SnO_2_. However, as the case of carbon coking generally observed in CH_4_ reforming catalysis^50–53^, sudden deposition of solid carbon may block the surface of SnO_2_. However, considering that solid carbon would float on the surface of reduced molten Sn, coking would not be a severe issue in MR of SnO_2_.

An almost identical reduction process was observed on SnO_2_(110) (Fig. 2b). The notable difference is that the first O-CH_3_^*^ dehydrogenation on SnO_2_(110) is relatively slow compared to that on SnO_2_(100). Dissociation of a O-CH_2_^*^ described in S3 and S4 is energetically and kinetically similar with that on SnO_2_(100). Most importantly, SnO_2_(110) surface provides the easier H_2_O desorption pathways as presented in S2 and S3 (Δ*E* = 0.11 eV) and S5 and S6 (Δ*E* = 0.13 eV). Because several high-index facets could coexist on the surface of SnO_2_ particles or powders, we believe that our DFT-generated SnO_2_ reduction pathways would proceed in a bi-functional or a multi-functional manner: Dissociation of a O-CH_3_^*^ or a O-CH_2_^*^ groups and formation and desorption of H_2_O could occur in a different local area of SnO_2_.

From the molecular structure points, the carbon from CH_4_ cannot aggressively reduce SnO_2_ in the early phase of reduction. The hydrogen atoms of CH_4_ reduce Sn oxides first, and the carbon from CH_4_ subsequently reduces the Sn oxides.

CH_4_ reforming of CO_2_ (dry reforming, CH_4_ + CO_2_ → 2H_2_ + 2CO) and CH_4_ steam reforming (CH_4_ + H_2_O → 3H_2_ + CO) have been applied to produce a mixture of CO and H_2_, which is a feedstock for Fisher-Tropsch synthesis. In general, the molar ratio of H_2_ and CO in the CH_4_ reforming product gas varies between 1 (dry reforming) and 3 (steam reforming)^12,21,39,50^. In Fig. 1a, at less than R = 0.62, H_2_ and CO were minority gas-phase products. However, as SnO_2_ and SnO are consumed and the gas-phase reactions occur in oxygen-depleted condition between CH_4_ and pre-produced gas-phase molecules, the H_2_/CO ratio rapidly increases as a function of the amount of supplied CH_4_. The rapid increase of the H_2_/CO ratio is phenomenologically feasible because excess CH_4_ supplies H, which enters into the system. Moreover, the negative ΔG_r_ also drives the release of H atoms from H_2_O molecules in the form of H_2_ molecules.

Typically, CH_4_ dry reforming operates at high temperatures above 500 °C and is catalyzed by the transition metal or novel metal catalysts^54,55^. It is not clear whether molten liquid Sn in our system catalyzes the reaction described in Fig. 1c. A recent report by Wetzel and coworkers showed that molten Sn facilitates the thermal dissociation of CH_4_ and thus the formation of solid-state carbon and H_2_^56,57^. Considering that solid-state carbon and H_2_ were increasingly accumulating at greater than R = 2.0 (Fig. 1b), CH_4_ dissociation by molten Sn may be attributed to the rapid H_2_ and solid carbon formation.

**Figure 3:**
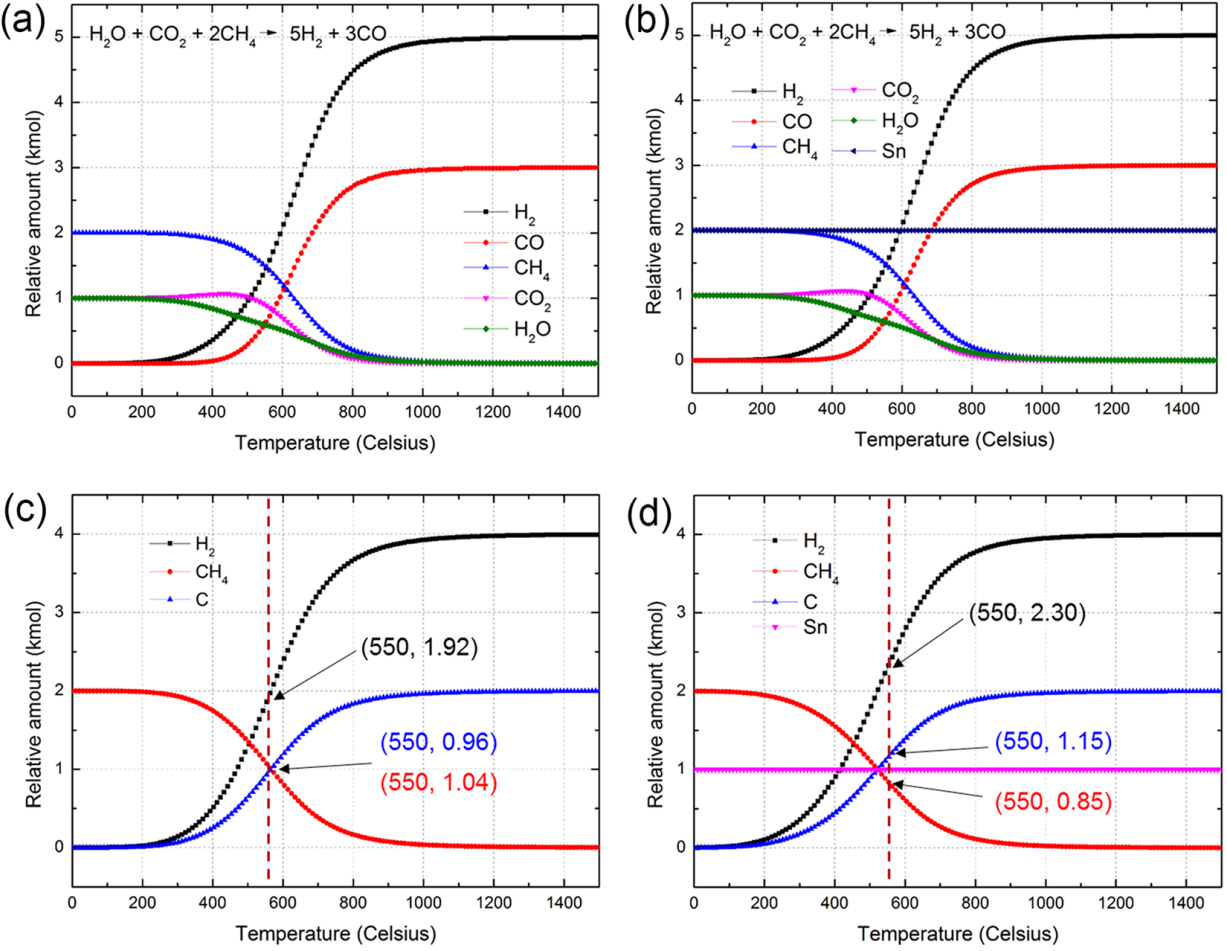
Thermodynamic simulations of the Sn effect on the gas phase reactions. (**a**,**b**) Temperature dependent equilibrium concentration of gas phase species without Sn (**a**) and with Sn (**b**). The presence of metallic Sn does not significantly affect the transformation of H_2_O + CO_2_ + 2CH_4_ to 5H_2_ + 3CO. (**c**,**d**) Temperature dependent concentration of CH_4_ and decomposed products; H_2_ and C, without Sn (**c**) and with Sn (**d**). Metallic Sn accelerates thermal decomposition of CH_4_ into C and H_2_. The numbers in the parentheses represent the equilibrium concentration of CH_4_, C, and H_2_ at 550 °C, at which theoretical maximum recovery of Sn was achieved (see Fig. 1d).

Thermodynamic simulation results presented in Fig. 3 show that the rate of the gas phase reaction, H_2_O + CO_2_ + 2CH_4_ → 5H_2_ + 3CO, does not critically affected by the presence of metallic Sn (or molten Sn, Fig. 3a and b). On the other hand, the addition of metallic Sn promotes thermal decomposition of CH_4_ (Fig. 3c and d). At 550 °C, at which the solid-state reduction is completed, the amount of decomposed CH_4_ was increased by 19.8 % in the presence of metallic Sn. This result theoretically reproduces the recent experimental findings reported by Wetzel and coworkers^56,57^.

Although the MR of SnO_2_ is not a catalytic reaction, a reasonable amount of the H_2_ and CO mixture was acquired as a byproduct as the R value exceeded 0.62. At approximately R = 2.0, H_2_O and CO_2_ in the system began to be depleted. All the oxygen in the system was taken by carbon-forming CO molecules, and the excess carbon from the CH_4_ was transformed to solid carbon. During this stage, the increasing H_2_ content in the system was entirely from the excess CH_4_.

### Experimental confirmation of CH_4_ reduction of SnO_2_

To verify the feasibility of the theoretically proposed concept of the MR of SnO_2_, we constructed a laboratory-scale experimental reduction furnace (Figure S1) with continuously flowing CH_4_ over the exposed SnO_2_ powders. Figure 3a and b show the before and after images of an aluminum boat initially loaded with 25 g of SnO_2_ and reduced at 1000 °C with supplied CH_4_. Ar-balanced CH_4_ gas continuously flowed through a quartz tube furnace for a total reduction time of 1 hour (flow rate of CH_4_: 250 sccm). Inevitably, a large portion of supplied CH_4_ bypasses SnO_2_ powders, being decomposed eventually into C and H_2_. Of course, an industrially applicable furnace should be designed to minimize the amount of bypassing CH_4_.

**Figure 4:**
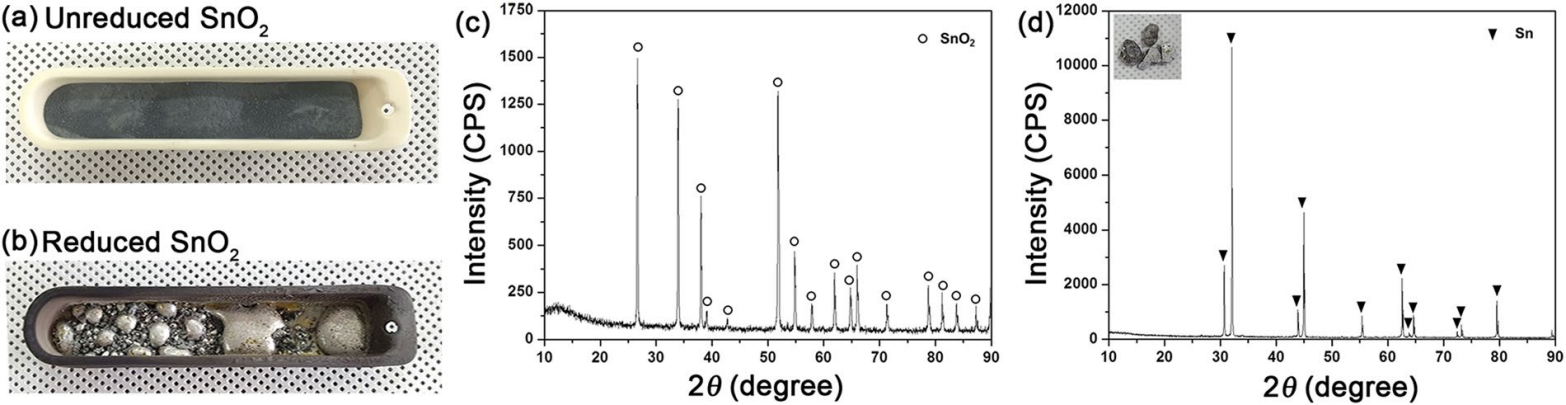
Experimental results of the CH_4_ reduction of SnO_2_. Photos of unreduced SnO_2_ powder (**a**) and CH_4_ reduced SnO_2_ (**b**). (**c**) and (**d**) show XRD spectra of unreduced SnO_2_ and reduced SnO_2_, respectively. XRD pattern in (d) demonstrates a typical case of *β*-Sn. The recovery rate of Sn was 79.9 %.

The presence of a glittering metallic phase in Fig. 3b shows that SnO_2_ was reduced to metallic Sn. An XRD analysis confirmed that initial SnO_2_ was reduced to crystalline *β*-Sn (Fig. 4c). A data set tabulated in Table 1 shows the high purity of the Sn reduced by CH_4_. It is remarkable that almost 80% of the supplied Sn was recovered (Fig. 4) even in the test batch experiment. Additionally, the reduced Sn had a high purity of 99.34% (ICP-analyzed). The concentration of the gas phase products shows a high H_2_/CO ratio of 5.99, which exceeds the theoretical maximum of conventional CH_4_ reforming (Table 2). As we mentioned above, thermally decomposed bypassing CH_4_ contributes to the high H_2_/CO ratio. The thermodynamically predicted H_2_/CO ratio in our reaction condition is about 2.30, which is close to that of convenient syngas for fuel production^58^.

**Table 1:** ICP-analyzed composition of the reduced Sn.

Element	Sn	Sb	As	Cu	Fe
Composition (at %)	99.34	0.13	0.001	0.49	0.04

**Table 2:** Molecular concentration of the gas-phase product from the CH_4_ reduction of SnO_2_ analyzed using gas chromatography^a^.

Molecule	H_2_^b^	CO^b^	CO_2_	CH_4_
Concentration (%)	83.9	14.0	1.7	0.4

^a^H_2_O was not analyzed.

^b^H_2_/CO = 5.99.

## Discussion

Development of an environmentally friendly and economically accessible reduction technology for low-quality used metal wastes is key for the sustainable use of limited resources. The MR of SnO_2_ method is environmentally and economically novel compared to the conventional dry- and wet-reduction methods, as the MR of SnO_2_ does not involve the use of solid reducing agents and liquid-phase acidic electrolytes. The efficiency of the MR of SnO_2_ was theoretically proposed and experimentally verified. Moreover, because the MR of SnO_2_ occurs at the solid-gas phase interface, the reaction can be more effective than solid-solid interactions of conventional dry reduction.

CH_4_ is a quite efficient and versatile reducing agent because the carbon and hydrogen of CH_4_ sequentially reduce SnO_2_ and produce various gas-phase products. This versatility of CH_4_ is highly beneficial for practical uses because the two most representative reducing agents, hydrogen and carbon, contribute to the total reducing power of CH_4_.

The theoretical interpretation predicts that as a result of a gas-phase reaction between excess CH_4_ and pre-produced H_2_O and CO_2_, the carbon and hydrogen in the reduction system are eventually transferred to CO and H_2_. Our theory-experimental combination study found that the H_2_/CO ratio in the gas-phase product is adjustable by controlling the amount of supplied CH_4_ and the temperature. Gas-phase reactions between initial gas-phase products of SnO_2_ and CH_4_ interaction should be considered for optimization of H_2_/CO ratio. The economic value added of the MR of SnO_2_ increases as long as syngas is producible as a byproduct. Industrially attractable quality of syngas could be produced through further optimization of the reaction temperature, total reaction time, and supplied SnO_2_/CH_4_ ratio.

In addition, our preliminary calculations show that butane or propane also vigorously reduce SnO_2_. For instance, we found that propane completed the SnO_2_ reduction at around 400 °C. Considering that the MR of SnO_2_ was finished at 550 °C (see Fig. 1c), this preliminary data predicts that the propane reduction of SnO_2_ would be economically more effective than the MR of SnO_2_. If their reducing power is verified by experiment, more economically accessible liquid natural gas or liquid petroleum gas could be applied for the reduction of SnO_2_. As a first step, we are currently working on the optimization of the MR of SnO_2_ and the utilization of liquid natural gas for SnO_2_ reduction. The relevant results will be reported in due course.

## Conclusions

We studied the mechanism of a novel MR of SnO_2_ which is a clean, environmentally friendly reduction method for SnO_2_. DFT calculations and thermodynamic simulations show that the carbon and hydrogen of CH_4_ bound to the surface of SnO_2_ independently and sequentially reduce SnO_2_. Various gas-phase products, such as H_2_O, CO, CO_2_, and H_2_, were produced from the early phase of reduction. The relative composition of the gas-phase product varies as a function of the amount of supplied CH_4_ and the temperature. In the early phase of the MR of SnO_2_ (low-temperature or less than R = 0.62 of supplied CH_4_), hydrogen acts as a dominant reducer. As the supplied CH_4_ increases, the carbon from CH_4_ aggressively takes the oxygen from H_2_O and CO_2_ forming CO. Hydrogen from CH_4_ and H_2_O is released as H_2_. The optimized operating condition of 1,000 °C and R = 2.0 was suggested from thermodynamic simulation data.

The reliability of this method was confirmed using an experimental batch test performed at 1,000 °C. The high recovery of our MR of SnO_2_ (approximately 80%) and the high purity (99.34%) of the reduced Sn demonstrate the novelty of our method.

Our results demonstrate a novel MR method for SnO_2_ as an efficient and green method for recovery of Sn from SnO_2_. In addition to the reduced metallic Sn, only several gas phase molecules and solid carbon were produced. The H_2_/CO ratio in the gas-phase product was controllable by the amount of CH_4_ supplied and the operating temperature. Our results open new avenues for the efficient and economic recovery of highly demanded metallic elements from complex oxide wastes, for example, the recovery of In and Sn from indium-tin oxide.

## Methods

### Thermodynamic simulations

Thermodynamic simulations were performed with the HSC 6.0 code (Outotec Research, www.hsc-chemistry.com). The relative thermodynamic stability of various Sn, C, O, and H chemical compounds was estimated at temperatures between 0 °C and 1,500 °C. For the initial equilibrium simulation, a mole of SnO_2_ was balanced with continuously increasing CH_4_ from 0 to 5 moles to clarify the effect of the SnO_2_/CH_4_ ratio (R) on the relative amount of the final products.

### Density functional theory calculations

Quantum chemical DFT calculations were performed with the VASP code^59^. A 3 × 2 × 4 rutile (110) and a 2 × 3 × 5 (100) supercells were used for surface reaction calculations and the most bottom triple layer was fixed during the optimization to ensure the structural robustness of the slab models (refer to Figure S2 for supercell geometry). Electron exchange and correlation were modeled using the Perdew-Burke-Ernzerhof (PBE)^60^ functional and the interaction between the ionic cores and the valence electrons was described with the projector augmented-wave method^61^. The valance-electron wave functions were expanded in the plane-wave basis set up to the energy cutoff of 400 eV. The convergence criteria for the electronic structure and the atomic geometry were 10^−4^ eV and 0.03 eV/Å, respectively.

### Experimental procedure

A high purity SnO_2_ electrode, which was previously used in glass-producing electric furnaces^32^, was acquired from Corning precision materials (Gumi, Korea). The average particle diameter of SnO_2_ powders was 135 μm (Figure S3). For the laboratory scale MR of SnO_2_ experiments, an alumina boat was loaded with 25 g of SnO_2_ powder and exposed to a stream of CH_4_ and Ar for 1 hour at 1000 °C. The CH_4_ flow rate was 250 sccm. The molar ratio of the total supplied CH_4_ was 1.85 to SnO_2_. The gas-phase concentration and composition of the reduced Sn was analyzed using gas chromatography (GC) and induced coupled plasma (ICP).

### Data availability

The datasets generated during and/or analyzed during the current study are available from the corresponding author on reasonable request.

## Electronic supplementary material

Supplementary information
