# Supplementary material for: Design of Reduction Process of SnO2 by CH4 for Efficient Sn Recovery
Source: Sci Rep. 2017 Oct 31;7:14427. doi: 10.1038/s41598-017-14826-7 (PMC5663740; doi:10.1038/s41598-017-14826-7)
Supplement: Supplementary file 1 — Supplementary information [file 41598_2017_14826_MOESM1_ESM.pdf]

Supplementary Information

Design of Reduction Process of SnO_2 by CH_4 for Efficient Sn Recovery

Hyunwoo Ha[1,†], Mi Yoo[1,†], Hyesung An[1,†], Kihyun Shin[2], Taeyang Han[1], Youhan Sohn[1], Sangyeol Kim[1,3], Sang-Ro Lee[3], Jun Hyun Han[1,*], and Hyun You Kim[1,*]

[1]Department of Materials Science and Engineering, Chungnam National University

99 Daehak-ro, Yuseong-gu, Daejeon 34134 Republic of Korea

[2]Department of Materials Science and Engineering,

KAIST, 291-Daehak-ro, Yuseong-gu, Daejeon, 34141 Korea

[3]A1 Engineering Co.,Ltd.,

80-19 Yulchonsandan 1-ro, Haeryong-myeon, Suncheon-si, Jeollanam-do, 58034

Republic of Korea

[†] These authors contributed equally to this work

To whom correspondence should be addressed: Prof. Jun-Hyun Han (jhhan@cnu.ac.kr), Prof. Hyun You Kim (kimhy@cnu.ac.kr)

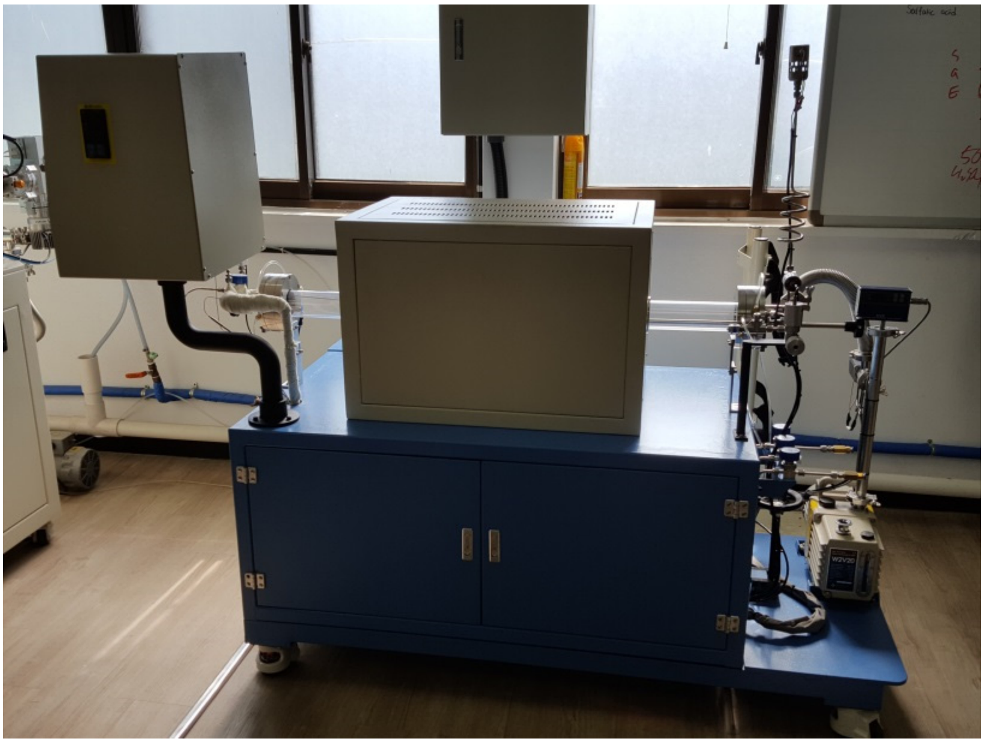

Figure S1. Reduction quartz tube furnace used for experimental study.

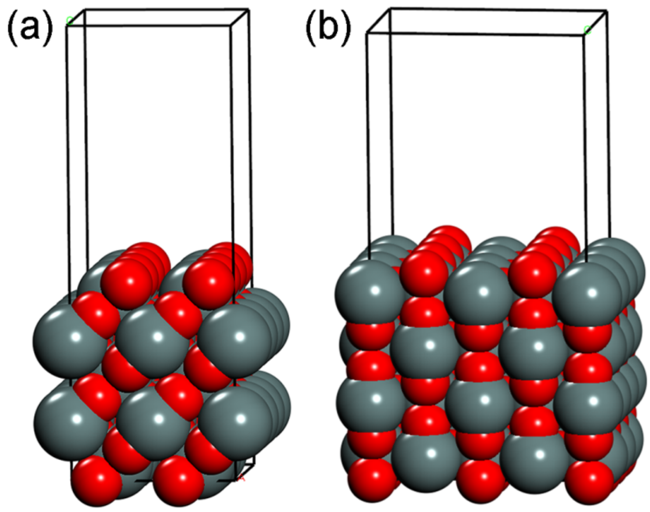

Figure S2. SnO_2 supercells used for DFT calculations: (a) SnO_2-(100) and (b) SnO_2-(110)

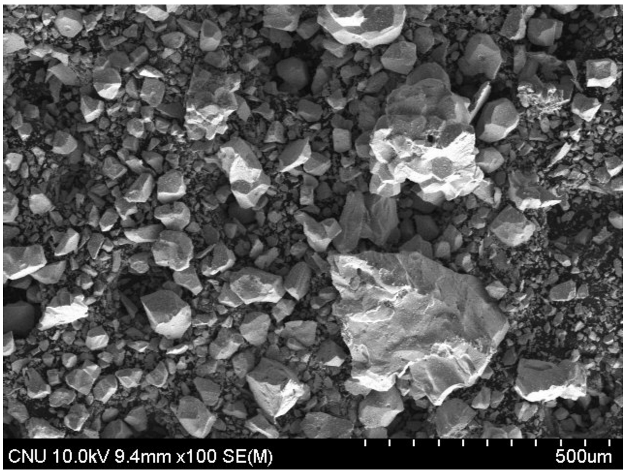

Figure S3. SEM image of SnO_2 powder. Average particle diameter was 135 μm (150 nm ~ 390 μm).
